# SATB1 and p16 Expression and Prognostic Value in Croatian Hodgkin Lymphoma Patients: A Unicentric Study

**DOI:** 10.3390/cells13161323

**Published:** 2024-08-08

**Authors:** Lučana Vicelić Čutura, Milan Vujčić, Davor Galušić, Viktor Blaslov, Marija Petrić, Antonija Miljak, Mirela Lozić, Benjamin Benzon, Katarina Vukojević, Toni Bubić, Nenad Kunac, Danijela Zjačić Puljiz, Ivana Kristina Delić Jukić, Marinela Križanac, Bernarda Lozić

**Affiliations:** 1 Department of Internal Medicine, Division of Haematology, University Hospital of Split, 21000 Split, Croatia; lucana.vicelic@gmail.com (L.V.Č.); milan.vujcic7@gmail.com (M.V.); marija.nuic21@gmail.com (M.P.);; 2 Department of Biochemistry and Medical Chemistry, University of Split School of Medicine, 21000 Split, Croatia; 3 Department of Anatomy Histology and Embryology, University of Split School of Medicine, 21000 Split, Croatia; 4 Laboratory of Morphology, Department of Histology and Embryology, School of Medicine, University of Mostar, 8800 Mostar, Bosnia and Herzegovina; 5 Faculty of Health Studies, University of Mostar, 88000 Mostar, Bosnia and Herzegovina; 6 Department of Anatomy, University of Mostar, 88000 Mostar, Bosnia and Herzegovina; 7 Center for Translational Research in Biomedicine, University of Split School of Medicine, 21000 Split, Croatia; 8 Department of Pathology, Judicial Medicine, and Cytology, Division of Pathology, University Hospital of Split, 21000 Split, Croatia; tonibubic15@gmail.com (T.B.);; 9 Department of Internal Medicine, Division of Nephrology and Haemodialysis, University Hospital of Split, 21000 Split, Croatia; 10 Department of Pediatric Disease, Division of Haematology, Oncology, Clinical Immunology and Genetics, University Hospital of Split, 21000 Split, Croatia; makrizanac@kbsplit.hr (M.K.); blozic@kbsplit.hr (B.L.); 11 University of Split School of Medicine, 21000 Split, Croatia

**Keywords:** SATB1, p16, Hodgkin lymphoma

## Abstract

Hodgkin lymphoma (HL) is a rare lymphoid neoplasm in which Hodgkin/Reed–Stenberg (HRS) cells are admixed with a population of non-neoplastic inflammatory cells and fibrosis. Dysregulated expressions of cell cycle regulators and transcription factors have been proven as one of the hallmarks of HL. In that context, SATB1 and p16 have been reported as potential regulators of HL progression and survival. However, to date, no studies have assessed the expression levels of SATB1 and p16 in HL in Croatian patients or their prognostic values. Therefore, we investigated the expression pattern of SATB1 and p16 in paraffin-embedded lymph node biopsies using standard immunohistochemistry. We found that 21% of the patients stained positive for SATB1, while 15% of the patients displayed positive staining for p16. Furthermore, we aimed to understand the prognostic value of each protein through the analysis of the overall survival (OS) and progression-free survival (PFS). SATB1 showed a significantly positive correlation with better OS and PFS, while p16 expression had no impact. Interestingly, when patients were stratified by a combination of the two studied markers, we found that patients in the SATB1+/p16- group tended to have the best prognosis in HL, according to statistical significance. In conclusion, SATB1 and p16 might be potentially useful as diagnostic and prognostic markers for HL.

## 1. Introduction

Hodgkin lymphoma (HL) is a rare monoclonal lymphoid neoplasm in which Hodgkin/Reed–Stenberg (HRS) cells are admixed with a population of non-neoplastic inflammatory cells and fibrotic tissue [1]. In most cases of classical Hodgkin’s lymphoma (cHL), HRS cells are derived from mature B cells that have lost most of their B cell phenotype [2]. They typically express cluster of differentiation 30 (CD30) in almost 100 percent of HL cases and CD15 in the majority of cases, while leukocyte common antigens CD45 and CD3 are typically not expressed [3]. Additionally, expression of common B cell antigens (CD20, CD79a, CD19) is typically absent [4,5]. RSs (Reed–Sternberg cells) are responsible for producing various cytokines that are crucial for enhancing the surrounding reactive infiltrate. Reactive cells can also produce cytokines that contribute to the survival and proliferation of malignant cells [6]. The two distinct entities of HL include the following: cHL and nodular lymphocyte-predominant HL (NLPHL; called nodular lymphocyte predominant B cell lymphoma in ICC) [7]. To date, the available treatments for HL can attain long-term disease control in most patients. However, 5–10% of patients will have refractory disease after initial therapy and about 10–30% will relapse [8]. Those patients represent a therapeutic challenge in hematology. Therefore, there is a need for better risk stratification to identify patients who are unlikely to respond well to standard therapeutic approaches. Additionally, the need to determine even more precise prognostic factors drives a tendency to reduce the toxicity of treatments. A better understanding of the pathogenesis of HL could lead to the development of new therapeutic targets. Overall, 1–10% of HRS cells in a tumor are incorporated within the background of the inflammatory infiltrate, making them difficult to detect [9]. So far, it has been shown that the majority of HRS cells are in the G1, S, or G2 phase of the cell cycle with impaired cell cycle and apoptosis regulation [4].

Special AT-rich sequence-binding protein-1 (SATB1) is a transcriptional regulator and genome organizer with the ability to regulate hundreds of genes, for which interest has increased in recent years as a tumor marker [10]. The expression of SATB1 has an important role in the development of T-cells [11]. Dysregulated SATB1 expression was shown to be involved in the pathogenesis of T-cell lymphoma [12]. Many studies have shown that loss of SATB1 expression is linked to the progression of mycosis fungoides, and moderate to high SATB1 expression is associated with a better prognosis in cutaneous T-cell lymphoma [13]. One study showed that aberrant SATB1 overexpression occurs in the majority of cutaneous CD30-positive lymphoproliferative diseases (CD30+ LPDs), which are characterized by the presence of CD30+ anaplastic large T cells [14]. Wang and colleagues demonstrated in their research that SATB1 overexpression leads to p21 silencing, making its expression inversely associated with SATB1 overexpression. SATB1 inhibits cell cycle inhibitor p21 expression by directly binding to the specific locus of *CDKN1A* promoter and contributes to disease progression [14]. However, SATB1 is highly expressed in many malignancies and linked to poor prognosis, distant metastasis, and an aggressive phenotype [11]. The expression of SATB1 in lymphoma remains unclear and contradictory [15]. Recently, a study showed that SATB1 overexpression is an independent factor for overall survival (OS) and progression-free survival (PFS) in patients with diffuse large B cell lymphoma (DLBCL) [16]. So far, no studies on the impact of SATB1 on the OS or PFS have been performed in HL.

P16 is known as a tumor suppressor protein and is a member of the Cyclin-Dependent Kinase (CDK) inhibitor family. Its well-known and important role as a negative regulator of the cell cycle involves blocking the G1 phase and preventing progression to the S phase [17]. Different studies have described a connection between the loss of p16 and patient outcomes in those with HL. For example, Calio and colleagues showed that higher levels of p16 and p21 are independent prognostic factors and have important protection against relapse [18]. On the other hand, overexpression of p16 has been described in malignant tumors, showing uncontrolled cellular proliferation, which can be explained by the negative feedback loop between retinoblastoma protein (pRB) and p16INK4a [19].

p16 positively regulates the basal expression of p21 in various human cell types [18]. p16 binds to CDK4 and CDK6, leading to the hypophosphorylation of pRB, which, in turn, halts the cell cycle at the G1/S phase. In the case of HL, loss of RB has been shown to be a poor prognostic factor [20].

Taken together, the currently available literature on the roles of SATB1 and p16 in lymphoma provides contradictory data.

However, to date, no study on HL has investigated the connection between p16 and SATB1. In our current retrospective study, we investigated the expression patterns of SATB1 and p16 and their connection with clinicopathological data as well as their potential prognostic value in HL for the first time by means of immunohistochemical staining of paraffin-embedded lymph node biopsies from a single institution. We hypothesized that a favorable prognosis in HL is associated with overexpression of SATB1 and loss of p16 expression, which may contribute novel insights into the cell cycle regulation of HRS cells

## 2. Materials and Methods

### 2.1. Tissue Samples of Patients

A total of 86 valid archived formalin-fixed, paraffin-embedded lymph node biopsies, previously histologically confirmed as HL, were included in this retrospective study. These lymphoma biopsies were retrieved from the archives of the Department of Pathology, Forensic Medicine and Cytology, University Hospital of Split, and were diagnosed between 2008 and 2018. The diagnoses of these lymphoma cases were established and classified according to the World Health Organization by a hematopathologist [1].

These 86 lymphoma cases were classified as cHL and NLPHL. cHL was also subdivided into subtypes as follows: nodular sclerosis (NS) (*n* = 60) and mixed cellularity (MC) (*n* = 20). Since we had only a few Lymphocyte-Rich (LR) (*n* = 4) and Lymphocyte-Depleted (LD) *(n* = 1) HL, we grouped these together with one case of nodular lymphocyte-predominant HL under the category “others” (Table 1). These 86 lymphoma cases included 39 males (45.3%) and 47 females (54.7%), aged 18 to 86 years. In the present study, all patients included had not received chemotherapy or radiotherapy for any other malignant disease prior to their diagnosis of Hodgkin’s lymphoma. The information about the patients’ clinical and laboratory characteristics as well as treatment outcomes was obtained from hospital records. In this study, the exclusion criteria included a previously diagnosed malignant disease and incomplete medical documentation. The study protocol was approved by the Ethics Committee of the University Hospital Split (2181-147-01/06/M.S.-19-2). All procedures were performed in accordance with the Helsinki Declaration.

Patients were staged according to the Ann Arbor staging system [21]. Pretreatment evaluation included clinical examination, laboratory tests, imaging (chest/abdomen CT), bone marrow evaluation, and echocardiograms for the evaluation of cardiac function. It should be noted that recently, staging has also been performed by PET/CT. We also estimated each patient’s fitness for treatment according to the ECOG score [22]. Bulky disease was defined as an abdominal or mediastinal mass ≥10 cm or greater than one-third of the internal transverse diameter of the thorax. For early-stage disease (I/II), we used prognostic factors EORTC (European Organization for the Research and Treatment of Cancer) and GHSG (German Hodgkin’s Study Group) [23]. For advanced-stage disease (III/IV), we used the international prognostic score (IPS) [24]. The patients were treated with chemotherapy protocols in the first line (ABVD, escBEACOPP, COPP, MOPP) with or without field radiotherapy, depending on the assessed stage of the disease, ECOG score, and comorbidities. Response assessment was defined as complete remission (CR) with no clinical, physical, or radiological evidence of disease. Partial disease (PR) was defined as a reduction of at least 50% measurable lesions with no increase in the size of other nodes, liver, or spleen. Progressive disease or treatment failure was defined as an increase in any measured lesions or the appearance of new lesions [18]. Clinical follow-up after therapy was conducted at intervals of every 3 months for the first two years, then every 6 months for the following 3 years, and thereafter, annually. During each visit, a detailed medical history was taken (constitutional symptoms, pruritus, lymphadenopathy, dyspnea, wheezing), and a physical examination was conducted to exclude relapse or treatment-related complications, especially in the first three to five years after therapy.

### 2.2. Immunohistochemical Staining

The tissue blocks were cut into 4 µm sections. Immunohistochemical slides were fixed for 30 min on a hot plate at 60 degrees Celsius. The entire immunohistochemical procedure was performed on the Ultra Benchmark (IHC/ISH Staining Module, Ventana, Tucson, AZ, USA) according to the manufacturer’s instructions. The specified procedures included deparaffinization, boiling in ultra CC1 buffer, antibody incubation, using the detection kit (UltraView Universal DAB Detection Kit, Ultra Benchmark, Tucson, Arizona, USA), and counterstaining with hematoxylin. Sections from tissue blocks were immunohistochemically analyzed with the following primary antibodies: p16 INK 4a (clone JC8; Santa Cruz Biotechnology; dilution 1:100) and SATB1 (clone C6; Santa Cruz Biotechnology; dilution 1:100). The quantitative evaluation of p16 and SATB1 in HRS was performed under a light microscope (Olympus BX46, Tokyo, Japan).

Human cervical carcinoma was used as a positive control for p16. We considered HRS cells positive for p16 if there was staining in the cytoplasm, nucleus, or both. Evaluation for the presence of positive expression of p16 (INK4a) was scored in the following way: negative (score 0), destitute of expression or in less than 10% of the positive HRS cells; low (score 1), 10% to 29% positive HRS cells; moderate (score 2), 30% to 59% of positive HRS cells; and high (score 3), ≥60% of positive HRS cells [18].

The positive control for SATB1 was the reactive microenvironment (lymphocytes). We considered HRS cells positive for SATB1 if there was staining in the cytoplasm, nucleus, or both. To evaluate SATB1 expression, we utilized the Immunoreactive Score (IRS) by Stenger, which was obtained by multiplying the staining intensity (SI) by the percentage of positive cells (PP) [25,26]. The staining intensity scoring of HRS-positive cells was performed as follows: negative—0 points; weak (pale yellow)—1 point; moderate (yellow-brown)—2 points; and strong (dark brown)—3 points. Positive HRS cells were scored as follows: 0–9% is 1 point; 10–50% is 2 points, and >50% is 3 points. If the result obtained by multiplying the two mentioned scores was 1 or lower, it represented negative SATB1 expression or reduced/absent expression. If the result was greater than 1, it represented positive SATB1 expression.

To evaluate the HRS expression of SATB1 and p16, ten microphotographs of random fields were taken under a light microscope (magnification 40×) for each sample. A pathologist examined the tissue blocks and was blinded to the patients’ clinical, laboratory, and pathological data.

### 2.3. Transcriptomic Analysis

Data for transcriptomic analysis were obtained from the Gene Expression Omnibus [GEO] database, GEO Accession viewer (nih.gov) [27]. They were uploaded to the Gene Pattern web suite (GenePattern, [28]) with the GEO importer tool for further analysis. To define genetic signatures, the Molecular signature database with its Gene ontology sets (C5) (GSEA | MSigDB | Browse Human Gene Sets (gsea-msigdb.org, [29]) was used. Furthermore, single-sample Gene Set Enrichment Analysis (GSEA) [30] with rank normalization was performed with the ssGSEA tool; its output was further statistically analyzed, as described in the next subsection.

### 2.4. Statistical Methods

Categorical data are presented as fractions (%) and counts; continuous data are presented as medians and interquartile ranges. Time-to-event data were modeled with an exponential decay-to-plateau equation (S(t)—survival probability at time t, S(t 0)—survival probability at the beginning of this study, S(P)—survival probability plateau; and the k–rate constant from which half-life can be calculated). To determine whether the expression of the studied markers could stratify the studied cohort, a null model in which a single survival curve describes all subpopulations was compared to the model in which each subpopulation is described by its own survival curve. Model comparison was performed by the Akaike information criterion (AIC), the F test, and standard model diagnostic tools. Uncertainty in parameter estimation is expressed as standard error (i.e., 68% confidence interval). To quantify the correlation between markers and preselected gene expression signatures, Reduced Major Axis (RMA) regression was used. GraphPad Prism 10 (GraphPad Software, Boston, MA, USA) and Past4 [31] were used.

## 3. Results

A total of 86 valid archived formalin-fixed, paraffin-embedded lymph node biopsies, previously histologically confirmed as HL, were included in this retrospective study (Table 1). The male-to-female ratio was roughly equal, and the median age was 41 years. Most of the patients had stage II disease, and the most common histological type was nodular sclerosis. More than half of the patients had B symptoms, while roughly a fifth of the patients had extranodal or bulky disease, and three-quarters of the patients had an ECOG functional score of 0. Roughly speaking, 80% of the patients were treated with the ABDV protocol, 10% with the COPP protocol, and 5% with some form of BEACOPP; the remaining 5% of the patients were treated with some combination of these three protocols. In addition to chemotherapy, 42% of the patients received radiation treatment. The median follow-up time was 50 months, and the range was from 1 to 197 months.

### 3.1. SATB1 Expression Associated with a More Favorable Prognosis

**Figure 1:**
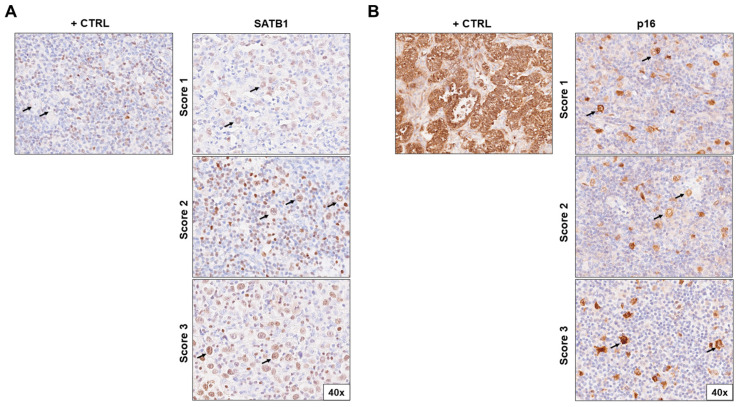
Immunohistochemical staining of (**A**) SATB1 and (**B**) p16 in lymph node biopsies of patients with Hodgkin lymphoma according to positivity score (1–3). The positive controls for SATB1 were surrounding lymphocytes; for p16, they were cervix adenocarcinoma cells. Pictures were taken at 40× magnification. Arrows indicate HRS cells. Abbreviations: +CTRL, positive control.

**Figure 2:**
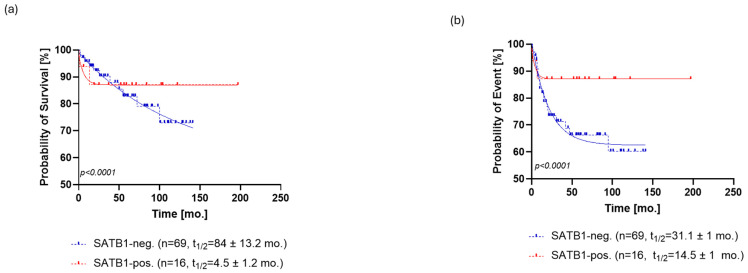
Kaplan–Meier analysis of overall survival (OS) for SATB1 (**a**) and p16 (**b**). (**a**) The OS of SATB1-positive patients was characterized by a population half-life of 4.5 ± 1.2 months and a survival plateau of 87 ± 1%. The SATB1-negative group demonstrated a much longer half-life of 84 ± 13.2 months; however, a survival plateau was not observed during the follow-up; the last survival probability observed was 72% after 12 years of follow-up. (**b**) Patients positive for p16 staining showed a half-life of 1.4 ± 0.77 months and a survival probability plateau of 75 ± 1.3%. Patients who were negative for p16 exhibited a half-life of 58 ± 6.1 months and the same survival plateau probability of 71 ± 1.4%. Abbreviations: neg., negative; pos., positive, mo., month.

**Figure 3:**
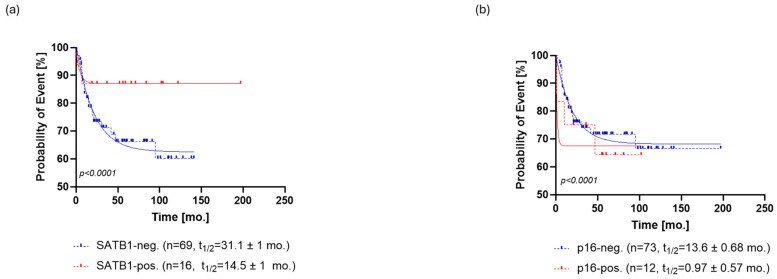
Kaplan–Meier analysis of progression-free survival (PFS) for SATB1 (**a**) and p16 (**b**). (**a**) SATB1-positive patients relapsed at a rate characterized by a half-life of 14.5 ± 1 months and a probability of relapse settled at the plateau of 87 ± 1%. On the other hand, SATB1-negative patients relapsed at a slower rate with a half-life of 31.1 ± 1 months, eventually reaching a plateau of 62 ± 0.5% (**b**) p16-positive patients exhibited a faster rate of relapse (PFS half-life = 0.97 ± 0.57 months) than the p16-negative patients (PFS half-life = 13.6 ± 0.68 months). However, the probability of relapse settled to an almost identical plateau in both groups, that is, 68 ± 0.5% for p16- patients and 67 ± 2% for p16+ patients. Abbreviations: neg., negative; pos., positive, mo., month.

SATB1 expression was positive in 21% (*n* = 16) patients (Figure 1A), and the level of SATB1 expression was ranked by the previously described score. SATB1 expression was able to stratify patients in terms of overall survival (OS, *p* < 0.0001, ΔAIC = 148.9, ER > 100, Figure 2a) and progression-free survival (PFS, *p* < 0.0001, ΔAIC = 194.5, ER > 100, Figure 3a). The OS of SATB1-positive patients was characterized by a population half-life of 4.5 ± 1.2 months and a survival plateau of 87 ± 1% (Figure 2a). The SATB1-negative group demonstrated a much longer half-life of 84 ± 13.2 months; however, a survival plateau was not observed during the follow-up; the last survival probability observed was 72% after 12 years of follow-up. On the other hand, extrapolation from the exponential model with plateau predicted the plateau at 58 ± 0.4% of survival probability.

Regarding PFS, SATB1-positive patients relapsed at a rate characterized by a half-life of 14.5 ± 1 months and a probability of relapse settled at the plateau of 87 ± 1%. On the other hand, SATB1-negative patients relapsed at a slower rate with a half-life of 31.1 ± 1 months, eventually reaching a plateau of 62 ± 0.5% probability of relapse (Figure 3a).

SATB1 expression did not stratify patients in any other clinical or pathological variable (Table S1). Likewise, it did not correlate with response to the first line of chemotherapy (Table S1).

### 3.2. p16 Expression Does Not Seem to Change Long Term Prognosis

Overall, 15% [*n* = 12] of the patients’ samples stained positively for p16 (Figure 1b). Expression of p16 could also stratify patients in terms of OS (*p* < 0.0001, ΔAIC = 109.3, ER > 100) and PFS (*p* < 0.0001, ΔAIC = 30.65, ER > 100) (Figure 2b and Figure 3b). A subpopulation of patients positive for p16 staining showed a half-life of 1.4 ± 0.77 months and a survival probability plateau of 75 ± 1.3%. The subpopulation of patients who were negative for p16 exhibited a half-life of 58 ± 6.1 months and the same survival plateau probability of 71 ± 1.4% (Figure 2b).

Similar to OS, p16-positive patients exhibited a faster rate of relapse (PFS half-life = 0.97 ± 0.57 months) than the p16-negative patients (PFS half-life = 13.6 ± 0.68 months). However, the probability of relapse settled to an almost identical plateau in both groups, that is, 68 ± 0.5% for p16- patients and 67 ± 2% for p16+ patients (Figure 3b).

Similar to SATB1, p16 expression did not stratify patients in any other clinical or pathological variable (Table S2). It did not correlate with response to the first line of chemotherapy (Table S2).

### 3.3. SATB1+/p16- Patients Have the Most Favorable Prognosis

**Figure 4:**
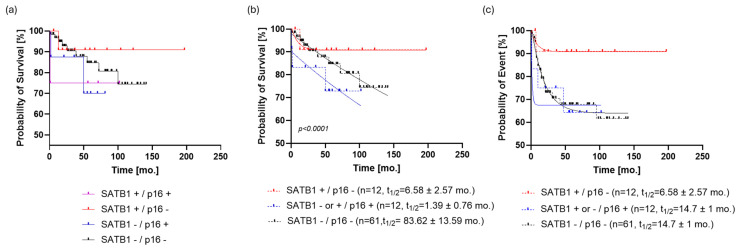
Stratification of the patient cohort by SATB1 and p16. (**a**) The SATB1+/p16- group (*n* = 12) of patients seems to have the best prognosis. (**b**) Dividing the study population into three subpopulations based on SATB1 and p16 expression resulted in a more parsimonious and accurate model than the one that ignored such information regarding OS (*p* < 0.0001, ΔAIC = 147.9, ER > 100). SATB1+/p16- patients reached the survival plateau of survival probability at 90.7 ± 0.54%; on the other hand, SATB1-/p16- patients had the last observed survival probability of 74% and a predicted, but not observed, survival plateau at 60 ± 0.5%. (**c**) If PFS was analyzed in the same way, it turned out that SATB1-/p16- and p16+ subpopulations follow the same dynamic (*p* = 0.7, ΔAIC = −9.672, ER = 1/126), with a half-life of 14.7 ± 1 months and a plateau of 63.4 ± 0.7%. On the other hand, the PFS curve of the SATB1+/p16- population was almost identical to the OS curve of the same group. Abbreviations: mo., month; AIC, Akaike information criterion; OS, overall survival; PFS, progression-free survival; ER, evidence ratio.

When the patients were stratified by a combination of the two studied markers, it was shown by graphical data analysis that the SATB1+/p16- group (*n* = 12) of patients seemed to have the best prognosis (Figure 4a). However, since the SATB1+/p16+ and SATB1-/p16+ groups had eight and four patients, respectively, we decided to combine them in a single p16+ group in order to proceed with the modeling of OS and PFS.

Dividing the study population into three subpopulations based on SATB1 and p16 expression resulted in a more parsimonious and accurate model than the one that ignored such information regarding OS (*p* < 0.0001, ΔAIC = 147.9, ER > 100). SATB1+/p16- patients reached the survival plateau of survival probability at 90.7 ± 0.54%; on the other hand, SATB1-/p16- patients had the last observed survival probability of 74% and a predicted, but not observed, survival plateau at 60 ± 0.5% (Figure 4b). Patients positive for p16 did not exhibit a clear survival plateau, i.e., models with or without a survival plateau were equally likely (*p* = 0.0761, ΔAIC = −0.03, ER = 1.02) (Table S3). Regarding event rates, p16+ patients had a half-life of 1.4 months, whereas those who were negative for both markers showed a half-life of 83.6 ± 12 months.

When PFS was analyzed in the same way, it turned out that the SATB1-/p16- and p16+ subpopulations followed the same dynamic (*p* = 0.7, ΔAIC = −9.672, ER = 1/126), with ta half-life of 14.7 ± 1 months and a plateau of 63.4 ± 0.7%. On the other hand, the PFS curve of the SATB1+/p16- population was almost identical to the OS curve of the same group (Figure 4c).

### 3.4. Analysis of Publicly Available Transcriptomic Datasets

**Figure 5:**
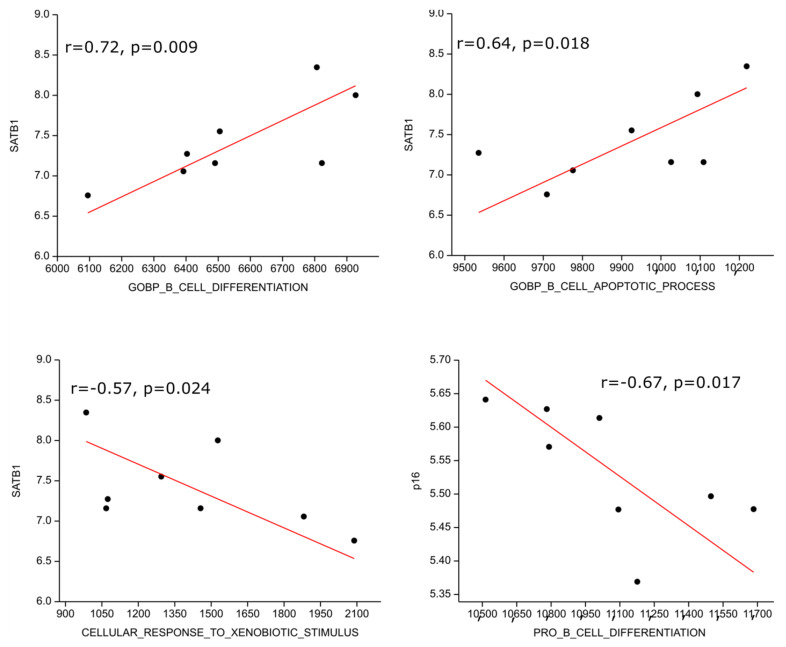
Correlation of genetic signatures with SATB1 and p16. The SATB1 transcript positively correlated with B cell differentiation, B cell apoptosis genetic signatures and inversely correlated with response to xenobiotic. P16 expression correlated inversely with pro B cell differentiation gene expression signature.

We performed gene set enrichment analysis on publicly available transcriptomic data from the NCBI GEO database and found one data series with eight HL patients (GSE120124, [32]). The SATB1 transcript highly positively correlated with B cell differentiation (r = 0.72; *p* = 0.009) and moderately with B cell apoptosis (r = 0.64; *p* = 0.018) genetic signatures; on the other hand, it moderately inversely correlated with response to xenobiotic (r = −0.57, *p* = 0.024), i.e., detoxification, genetic signatures. P16 expression correlated moderately inversely with the pro B cell differentiation gene expression signature (r = −0.67, *p* = 0.017) (Figure 5).

## 4. Discussion

Hodgkin lymphoma is a relatively rare monoclonal lymphoid neoplasm of HRS cells mixed with non-neoplastic inflammatory cells and fibrotic tissue. In cHL, HRS cells derive from mature B cells; however, they lose most of the markers of normal B cells. These cells virtually always express CD30 and usually express CD15, but they do not contain CD45, CD3, or common B-cell antigens such as CD20, CD79a, or CD19. HRS cells are capable of producing cytokines that fuel the adjacent reactive infiltrate, thereby nourishing the malignant cells [1,2,3,4,5,6].

HL is divided into the following categories: cHL and NLPHL. The treatments available at the moment offer long-term disease control in most cases to patients; however, 5–10% have refractory diseases, and 10–30% have a relapse, considered therapeutically challenging. In this regard, improved risk stratification and the identification of more precise prognostic factors are critically relevant. A better understanding of HL pathogenesis may enable new therapeutic targets and reduce treatment toxicity [7,8].

According to the available data, HL is most commonly diagnosed in younger populations [33], which is consistent with our study, where the median age of the patient was 41 years. In the present study, the male-to-female ratio in HL is approximately equal, which aligns with findings from other studies, especially considering that nodular sclerosis is the most common subtype [34]. In our study, the most common histological type was nodular sclerosis, which is consistent with its distribution in economically developed countries [20,33,35]. The majority of the patients were in stage II (42 patients, 48,8%). Similarly, Shamoon et al. and SEER (Surveillance, Epidemiology, and End Results program) reported that about half of the studied patients had stage II [36,37].

SATB1 is a nuclear protein involved in remodeling and folding chromatin mechanisms to control the expression of multiple genes, and it is primarily expressed in the CNS and thymocytes [38]. It is also known that SATB1 regulates the level of histone methylation and acetylation, which is important in differentiation and apoptosis. To date, studies have demonstrated the role of SATB1 in controlling genes associated with the immune response, cell proliferation, cell cycle, DNA repair, and various signaling pathways [13,38]. Until now, little was known about the potential role of SATB1 in HL.

The aim of this study was to investigate the expression of SATB1 and p16 in HL as well as to determine possible relationships with the clinicopathological data of patients and their value as survival indicators. In this context, we found that high SATB1 expression is associated with a more favorable prognosis. However, SATB1 was initially defined as an oncogene because it is overexpressed in many malignancies, including breast cancer, colorectal cancer, prostate cancer, liver cancer, bladder cancer, and ovarian cancer. In most cases, elevated levels of SATB1 in solid tumors are associated with poor prognosis, distant metastasis, and an aggressive phenotype [11,39]. However, certain studies have also demonstrated low levels of SATB1 in non-small cell lung tumors with a proven unfavorable forecast in lung squamous cell carcinomas [40]. The role and significance of SATB1 are opposite in solid tumors compared with hematological malignancies. Wang and colleagues demonstrated SATB1 deficiency in Sezary syndrome, an aggressive leukemic variant of cutaneous T-cell lymphoma (CTCL) involved in apoptosis resistance in tumor cells [41]. On the other hand, in a previous study, SATB1 was considered an oncogene in CD30+ cutaneous T-cell lymphomas because its activity was directed towards silencing p21 [14]. Also, Luo and colleagues concluded that SATB1 can serve as a predictor of a good response to therapy in acute myeloid leukemia (AML) [42]. It has been shown that a reduction in SATB1 expression enables cellular proliferation in AML through the activation of the NF-κB signaling pathway [42]. One study previously reported reduced expression of SATB1 in adult T cell leukemia (ATL), which could increase Jurkat cell invasiveness through the activation of the Wnt/β-catenin signaling pathway [38]. However, Yi and colleagues demonstrated contrasting results in DLBCL, where increased expression of SATB1 was found to be an independent prognostic indicator of shorter PFS and OS [16]. The observed discrepancy in results among hematologic malignancies can be explained by the different immunophenotypes of the malignant cells. Specifically, CD30 expression is limited to three hematologic diseases including cHL, anaplastic large cell lymphomas (ALCLs), and primary cutaneous CD30+ T-cell lymphoproliferative disorders in which SATB1 expression is associated with a better prognosis. In contrast, tumor cells in DLBCL express common B cell antigens (CD20, CD79a, CD19), while CD30 is expressed in only 25% of cases [43]. To our knowledge, our retrospective study is the first to associate SATB1 expression with the clinicopathological characteristics of HL patients and its impact on OS and PFS. In our study, a positive association between SATB1 and a favorable prognosis in HL was demonstrated. The mechanism by which SATB1 mediates HL is not known, but it is likely that multiple potential mechanisms exist, each requiring further investigation. This favorable association may stem from SATB1’s involvement in immune modulation and apoptosis [38,41]. Therefore, we postulate that SATB1 overexpression could enhance the immune system’s recognition of RS cells, leading to more effective immune-mediated destruction of the tumor. Additionally, SATB1 may promote apoptosis in RS cells, thereby reducing the overall tumor burden and improving patient outcomes.

One possible initial mechanism to investigate is the impact of reduced SATB1 expression in HRS cells on the activity of the NF-κB signaling pathway, a known regulator of apoptosis dysregulation in HL [44]. The second mechanism to consider is the influence of SATB1 on the anti-apoptotic marker BCL2, given the documented overexpression of BCL2 in HRS cells of patients with refractory and early relapsed cHL [45]. A third mechanism could be the influence of SATB1 on the expression of cytokines such as IL-5, IL-9, IL-8, CCL5, and CCL28 in the microenvironment of HL. Previous studies conducted in Mycosis fungoides and Sézary syndrome have shown that SATB1 can influence the expression of IL5 and IL9 [46]. Lastly, the impact of SATB1 on the presence of EBV and the expression of STAT3 could be considered, especially given the known overexpression of STAT3 in the presence of EBV [47].

Altogether, we could suggest a specific role of SATB1 in modulating gene expression patterns related to the immune response or cellular processes that influence HL pathogenesis. Additionally, SATB1’s involvement in regulatory networks that affect tumor microenvironment and immune cell interactions might contribute to its association with better outcomes in HL. Further investigation is needed to understand these mechanisms fully. It appears that SATB1’s role can vary depending on the specific type of cancer.

p16 is a well-known tumor suppressor protein that is involved in the regulation of the cell cycle and apoptosis. In our study, 15% of the samples from patients with HL were positively stained for p16. Irshad and colleagues reported that the expression of p16 occurs in 52.3% of HL cases and Zhao demonstrated the expression of the p16 protein in 71.7% of HL cases, which is more compared with our study [48,49]. In contrast to our study, Calio and colleagues demonstrated that patients with HL who have absent or low expression of p21 and p16 are prone to relapse or do not respond to therapy, as inactivation of tumor suppressor cellular pathways can lead to uncontrolled proliferation, along with the well-known role of EBV in inhibiting p16 expression [18]. However, p16 loss could disrupt the cell cycle in RS cells, rendering them more vulnerable to chemotherapy and other treatments, thus improving therapeutic efficacy. The variability in the expression of p16 in samples from patients with HL among different studies can be explained by the use of different immunohistochemical scoring methods.

The role of p16 in cellular senescence is well-known. It has been shown that increased expression of p16 in senescent cells acts as a cell cycle inhibitor, resulting in these cells being negative for Ki67, a proliferation marker [49]. We now know that HRS cells exhibit senescence characteristics that can have both anti-proliferative functions, limiting tumor growth, and pro-inflammatory functions by producing cytokines and chemokines that promote tumor progression [50]. However, overexpression of p16 is associated with poor prognosis in some solid tumors such as colon and breast cancer [51,52], highlighting the caution needed when using p16 as a prognostic marker. Increased expression of p16 can occur following the inactivation of the p16-RB signaling pathway, leading to loss of RB and creating a negative feedback loop. To date, it has been shown that loss of RB in HL is a poor prognostic factor that affects overall survival and achievement of complete remission [20]. Therefore, loss of RB could be one of the mechanisms in HL leading to overexpression of p16 [19,20]. Our current study suggests that p16, by itself, is not suitable as a survival marker. However, when combined with SATB1 overexpression, its loss indicates a favorable prognosis, which is consistent with the aforementioned study [19].

We performed gene set enrichment analysis on publicly available transcriptomic data from the NCBI GEO database. Although our search yielded data for only eight patients with HL, the investigated results are consistent with our findings. The SATB1 transcript positively correlated with B cell differentiation and B cell apoptosis genetic signatures, but it inversely correlated with response to xenobiotics. In contrast, p16 expression inversely correlated with the pro-B cell differentiation gene expression signature.

## 5. Conclusions

Our current study found that patients in the SATB1+/p16- group tend to have the best prognosis in HL. SATB1 and p16 immunohistochemical staining may serve as useful prognostic indicators, helping to identify patients with a favorable prognosis. It could help in the stratification of patients, allowing for more personalized treatment approaches. Furthermore, understanding the molecular mechanisms behind SATB1 overexpression and p16 loss in HL could open new avenues for targeted therapies. Modulating these pathways may enhance treatment responses and improve overall survival rates in HL. Further research is needed to confirm our results and potentially incorporate these antibodies into routine clinical practice.

## Figures and Tables

**Table 1:** Demographic and clinicopathological characteristics of HL patients [*n* = 86].

number of patients	86
age [years, median], IQR [years]	41, 28 to 55
sex [*n*, %]	
female	47, 54.7%
male	39, 45.3%
histological type [*n*, %]	
mixed cellularity	20, 23.3%
nodular sclerosis	60, 69.8%
other	6, 6.9%
clinical stage [*n*, %]	
I	9, 10.5%
II	42, 48.8%
III	16, 18.6%
IV	19, 22.1%
+ extranodal localization [*n*, %]	15, 17.4%
+ B symptoms [*n*, %]	54, 62.3%
+ bulky disease [*n*, %]	15, 17.4%
EORCT [for stages I and II, *n*, %]	
0	13, 25.5%
1	15, 29.4%
2	16, 31.4%
3	7, 13.7%
GHSG [for stages I & II, *n*, %]	
0	12, 23.5%
1	15, 29.4%
2	18, 35.3%
3	6, 11.7%
IPS [for stages III and IV, *n*, %]	
1	8, 22.9%
2	10, 28.6%
3	12, 34.3%
4	2, 5.7%
5	3, 8.6%
ECOG [*n*, %]	
0	64, 74.4%
1	18, 20.9%
2	4, 4.7%

Abbreviations: IQR, interquartile range; *n*, number of cases; EORCT, European Organization for the Research and Treatment of Cancer; GHSG, German Hodgkin’s Study Group; IPS, International Prognostic Score; ECOG, Eastern Cooperative Oncology Group.

## Data Availability

The data presented in this study are available on request from the corresponding author.

## Supplementary Materials

The following supporting information can be downloaded at: https://www.mdpi.com/article/10.3390/cells13161323/s1, Table S1: SATB1 expression level correlation with the clinicopathological characteristics of HL patients at diagnosis and response to the first line of chemotherapy; Table S2: p16 expression level correlation with the clinicopathological characteristics of HL patients at diagnosis and response to the first line of chemotherapy; Table S3: Statistical analysis of the correlation of clinicopathological characteristics and three subpopulations of patients based on SATB1 and p16 expression.

## References

[1] Alaggio R., Amador C., Anagnostopoulos I., Attygalle A.D., de Oliveira Araujo I.B., Berti E., Bhagat G., Borges A.M., Boyer D., Calaminici M. (2022). The 5th edition of the World Health Organization Classification of Haematolymphoid Tumours: Lymphoid Neoplasms. Leukemia.

[2] Liu Y., van den Berg A., Veenstra R., Rutgers B., Nolte I., van Imhoff G., Visser L., Diepstra A. (2013). PML Nuclear Bodies and SATB1 Are Associated with HLA Class I Expression in EBV+ Hodgkin Lymphoma. PLoS ONE.

[3] Harris N.L. (1999). Hodgkin’s disease: Classification and differential diagnosis. Mod. Pathol..

[4] Zukerberg L.R., Collins A.B., Ferry J.A., Harris N.L. (1991). Coexpression of CD15 and CD20 by Reed-Sternberg cells in Hodgkin’s disease. Am. J. Pathol..

[5] Schmid C., Pan L., Diss T., Isaacson P.G. (1991). Expression of B-cell antigens by Hodgkin’s and Reed-Sternberg cells. Am. J. Pathol..

[6] Skinnider B.F., Mak T.W. (2002). The role of cytokines in classical Hodgkin lymphoma. Blood.

[7] Campo E., Jaffe E.S., Cook J.R., Quintanilla-Martinez L., Swerdlow S.H., Anderson K.C., Brousset P., Cerroni L., de Leval L., Dirnhofer S. (2022). The International Consensus Classification of Mature Lymphoid Neoplasms: A report from the Clinical Advisory Committee. Blood.

[8] Voorhees T.J., Beaven A.W. (2020). Therapeutic Updates for Relapsed and Refractory Classical Hodgkin Lymphoma. Cancers.

[9] Al-Salam S., Awwad A., Sudhadevi M., Daoud S., Nagelkerke N.J.D., Castella A., Chong S.M., Alashari M. (2013). Epstein-Barr virus infection correlates with the expression of COX-2, p16(INK4A) and p53 in classic Hodgkin lymphoma. Int. J. Clin. Exp. Pathol..

[10] Jankowska-Konsur A., Kobierzycki C., Reich A., Grzegrzolka J., Bieniek A., Dziegiel P. (2016). Expression of SATB1, MTI/II and Ki-67 in Mycosis Fungoides. Anticancer Res..

[11] Glatzel-Plucińska N., Piotrowska A., Dzięgiel P., Podhorska-Okołów M. (2019). The Role of SATB1 in Tumour Progression and Metastasis. Int. J. Mol. Sci..

[12] Fredholm S., Willerslev-Olsen A., Met Ö., Kubat L., Gluud M., Mathiasen S.L., Friese C., Blümel E., Petersen D.L., Hu T. (2018). SATB1 in Malignant T Cells. J. Investig. Dermatol..

[13] Grzanka D., Gagat M., Izdebska M., Marszałek A. (2015). Expression of special AT-rich sequence-binding protein 1 is an independent prognostic factor in cutaneous T-cell lymphoma. Oncol. Rep..

[14] Wang Y., Gu X., Zhang G., Wang L., Wang T., Zhao Y., Zhang X., Zhou Y., Kadin M., Tu P. (2014). SATB1 overexpression promotes malignant T-cell proliferation in cutaneous CD30+ lymphoproliferative disease by repressing p21. Blood.

[15] Frömberg A., Engeland K., Aigner A. (2018). The Special AT-rich Sequence Binding Protein 1 (SATB1) and its role in solid tumors. Cancer Lett..

[16] Yi H., Wei Y., Chen J.-J. (2022). Expression of SATB1 is a prognostic indicator for survival in diffuse large B-cell lymphoma patients. Eur. Rev. Med. Pharmacol. Sci..

[17] Irshaid F., Tarawneh K., Alshdefat A., Dilmi F., Jaran A., Al-Hadithi R., Al-Khatib A. (2013). Loss of P16 Protein Expression and Its Association with Epstein-Barr Virus LMP-1 Expression in Hodgkin’s Lymphoma. Iran. J. Cancer Prev..

[18] Caliò A., Zamò A., Ponzoni M., Zanolin M.E., Ferreri A.J.M., Pedron S., Montagna L., Parolini C., Fraifeld V.E., Wolfson M. (2015). Cellular Senescence Markers p16INK4a and p21CIP1/WAF Are Predictors of Hodgkin Lymphoma Outcome. Clin. Cancer Res..

[19] Inoue K., Fry E.A. (2018). Aberrant expression of p16INK4a in human cancers—A new biomarker?. Cancer Rep. Rev..

[20] Morente M.M., Piris M.A., Abraira V., Acevedo A., Aguilera B., Bellas C., Fraga M., Garcia-Del-Moral R., Gomez-Marcos F., Menarguez J. (1997). Adverse clinical outcome in Hodgkin’s disease is associated with loss of retinoblastoma protein expression, high Ki67 proliferation index, and absence of Epstein-Barr virus-latent membrane protein 1 expression. Blood.

[21] Lister T.A., Crowther D., Sutcliffe S.B., Glatstein E., Canellos G.P., Young R.C., Rosenberg S.A., Coltman C.A., Tubiana M. (1989). Report of a committee convened to discuss the evaluation and staging of patients with Hodgkin’s disease: Cotswolds meeting. J. Clin. Oncol..

[22] Oken M.M., Creech R.H., Tormey D.C., Horton J., Davis T.E., McFadden E.T., Carbone P.P. (1982). Toxicity and response criteria of the Eastern Cooperative Oncology Group. Am. J. Clin. Oncol..

[23] Eichenauer D.A., Aleman B.M.P., André M., Federico M., Hutchings M., Illidge T., Engert A., Ladetto M., ESMO Guidelines Committee (2018). Hodgkin lymphoma: ESMO Clinical Practice Guidelines for diagnosis, treatment and follow-up. Ann. Oncol..

[24] Hasenclever D., Diehl V. (1998). A prognostic score for advanced Hodgkin’s disease. International Prognostic Factors Project on Advanced Hodgkin’s Disease. N. Engl. J. Med..

[25] Remmele W., Stegner H.E. (1987). Recommendation for uniform definition of an immunoreactive score (IRS) for immunohistochemical estrogen receptor detection (ER-ICA) in breast cancer tissue. Pathologe.

[26] Chen Z., Li Z., Li W., Zong Y., Zhu Y., Miao Y., Xu Z. (2015). SATB1 Promotes Pancreatic Cancer Growth and Invasion Depending on MYC Activation. Dig. Dis. Sci..

[27] Edgar R., Domrachev M., Lash A.E. (2002). Gene Expression Omnibus: NCBI gene expression and hybridization array data repository. Nucleic Acids Res..

[28] Reich M., Liefeld T., Gould J., Lerner J., Tamayo P., Mesirov J.P. (2006). GenePattern 2.0. Nat. Genet..

[29] Subramanian A., Tamayo P., Mootha V.K., Mukherjee S., Ebert B.L., Gillette M.A., Paulovich A., Pomeroy S.L., Golub T.R., Lander E.S. (2005). Gene set enrichment analysis: A knowledge-based approach for interpreting genome-wide expression profiles. Proc. Natl. Acad. Sci. USA.

[30] Barbie D.A., Tamayo P., Boehm J.S., Kim S.Y., Moody S.E., Dunn I.F., Schinzel A.C., Sandy P., Meylan E., Scholl C. (2009). Systematic RNA interference reveals that oncogenic KRAS-driven cancers require TBK1. Nature.

[31] Hammer Ø., Harper D.A.T., Ryan P.D. (2001). PAST: Paleontological statistics software package for education and data analysis. Palaeontol. Electron..

[32] Péricart S., Tosolini M., Gravelle P., Rossi C., Traverse-Glehen A., Amara N., Franchet C., Martin E., Bezombes C., Laurent G. (2018). Profiling Immune Escape in Hodgkin’s and Diffuse large B-Cell Lymphomas Using the Transcriptome and Immunostaining. Cancers.

[33] Munir F., Hardit V., Sheikh I.N., AlQahtani S., He J., Cuglievan B., Hosing C., Tewari P., Khazal S. (2023). Classical Hodgkin Lymphoma: From Past to Future—A Comprehensive Review of Pathophysiology and Therapeutic Advances. Int. J. Mol. Sci..

[34] Morton L.M., Wang S.S., Devesa S.S., Hartge P., Weisenburger D.D., Linet M.S. (2006). Lymphoma incidence patterns by WHO subtype in the United States, 1992–2001. Blood.

[35] Moscona-Nissan A., Mancilla-Osuna M.F., Bardán-Duarte A., Rendón-Macías M.E. (2023). Classical Hodgkin lymphoma histologic subtypes distribution among geographical regions and correlation with Human Development Index. Health Sci. Rev..

[36] Shamoon R.P., Ali M.D., Shabila N.P. (2018). Overview and outcome of Hodgkin’s Lymphoma: Experience of a single developing country’s oncology centre. PLoS ONE.

[37] Bazzeh F., Rihani R., Howard S., Sultan I. (2010). Comparing adult and pediatric Hodgkin lymphoma in the Surveillance, Epidemiology and End Results Program, 1988–2005: An analysis of 21,734 cases. Leuk. Lymphoma.

[38] Luo X.-D., Yang S.-J., Wang J.-N., Tan L., Liu D., Wang Y.-Y., Zheng R.-H., Wu X.-H., Xu L.-H., Tan H. (2015). Downregulation of SATB1 increases the invasiveness of Jurkat cell via activation of the WNT/β-catenin signaling pathway in vitro. Tumor Biol..

[39] Li Y., Wang J., Yu M., Wang Y., Zhang H., Yin J., Li Z., Li T., Yan H., Li F. (2018). SNF5 deficiency induces apoptosis resistance by repressing SATB1 expression in Sézary syndrome. Leuk. Lymphoma.

[40] Selinger C.I., Cooper W.A., Al-Sohaily S., Mladenova D.N., Pangon L., Kennedy C.W., McCaughan B.C., Stirzaker C., Kohonen-Corish M.R. (2011). Loss of Special AT-Rich Binding Protein 1 Expression is a Marker of Poor Survival in Lung Cancer. J. Thorac. Oncol..

[41] Wang Y., Su M., Zhou L.L., Tu P., Zhang X., Jiang X., Zhou Y. (2011). Deficiency of SATB1 expression in Sézary cells causes apoptosis resistance by regulating FasL/CD95L transcription. Blood.

[42] Luo X., Xu L., Wu X., Tan H., Liu L. (2019). Decreased SATB1 expression promotes AML cell proliferation through NF-κB activation. Cancer Cell Int..

[43] Hu S., Xu-Monette Z.Y., Balasubramanyam A., Manyam G.C., Visco C., Tzankov A., Liu W.-M., Miranda R.N., Zhang L., Montes-Moreno S. (2013). CD30 expression defines a novel subgroup of diffuse large B-cell lymphoma with favorable prognosis and distinct gene expression signature: A report from the International DLBCL Rituximab-CHOP Consortium Program Study. Blood.

[44] Jardin F. (2022). NFkB Pathway and Hodgkin Lymphoma. Biomedicines.

[45] Rassidakis G.Z., Medeiros L.J., Vassilakopoulos T.P., Viviani S., Bonfante V., Nadali G., Herling M., Angelopoulou M.K., Giardini R., Chilosi M. (2002). BCL-2 expression in Hodgkin and Reed-Sternberg cells of classical Hodgkin disease predicts a poorer prognosis in patients treated with ABVD or equivalent regimens. Blood.

[46] Herrera A., Fredholm S., Cheng A., Mimitou E.P., Seffens A., Bar-Natan M., Sun A., Latkowski J.-A., Willerslew-Olsen A., Buus T.B. (2020). Low SATB1 Expression Promotes IL-5 and IL-9 Expression in Sezary Syndrome. J. Investig. Dermatol..

[47] de la Cruz-Merino L., Lejeune M., Nogales Fernández E., Henao Carrasco F., Grueso López A., Illescas Vacas A., Pulla M.P., Callau C., Álvaro T. (2012). Role of immune escape mechanisms in Hodgkin’s lymphoma development and progression: A whole new world with therapeutic implications. Clin. Dev. Immunol..

[48] Irshaid F., Jaran A., Dilmi F., Tarawneh K., Hadeth R., Khatib A.A. (2010). Prevalence of Epstein-Barr Virus Latent Membrane Protein-1 in Jordanian Patients with Hodgkin’s Lymphoma and Non-Hodgkin’s Lymphoma. J. Biol. Sci..

[49] Zhao P., Lu Y., Liu L., Zhong M. (2008). Aberrant Expression of ID2 protein and its correlation with EBV-LMP1 and P16(INK4A) in Classical Hodgkin Lymphoma in China. BMC Cancer.

[50] Gopas J., Stern E., Zurgil U., Ozer J., Ben-Ari A., Shubinsky G., Braiman A., Sinay R., Ezratty J., Dronov V. (2016). Reed-Sternberg cells in Hodgkin’s lymphoma present features of cellular senescence. Cell Death Dis..

[51] Lebok P., Roming M., Kluth M., Koop C., Özden C., Taskin B., Hussein K., Lebeau A., Witzel I., Wölber L. (2016). p16 overexpression and 9p21 deletion are linked to unfavorable tumor phenotype in breast cancer. Oncotarget.

[52] Zhou N., Gu Q. (2018). Prognostic and clinicopathological value of p16 protein aberrant expression in colorectal cancer A PRISMA-compliant Meta-analysis. Medicine.

