# Supplementary material for: SATB1 and p16 Expression and Prognostic Value in Croatian Hodgkin Lymphoma Patients: A Unicentric Study
Source: Cells. 2024 Aug 8;13(16):1323. doi: 10.3390/cells13161323 (PMC11352626; doi:10.3390/cells13161323)
Supplement: Supplementary file 1 [file cells-13-01323-s001.zip › cells-3116967-supplementary.pdf]

Supplemental Material

SATB1 and p16 expression and prognostic value in Croatian Hodgkin lymphoma patients: a unicentric study

Table S1. SATB1 expression level correlation with clinicopathological characteristics of HL patients at diagnosis and response to first line of chemotherapy.

	**SATB1-**	**SATB1+**	**p value**
**number of patients**	70	16	
**age (years, median, IQR)**	36.5 (27.5 to 52)	50.5 (30 to 64.5)	0.242*
**sex (n, %)**			0.0117†
female	27, 38.6%	12, 75%	
male	43, 61.4%	4, 25%	
**histological type (n, %)**			0.4727†
mixed cellularity	16, 22.9%	4, 25%	
nodular sclerosis	50, 71.4%	10, 62.5%	
other	4, 5.7%	2, 12.5%	
**clinical stage (n, %)**			0.0928†
I	9, 12.9%	0, 0%	
II	42, 51.4%	6, 37.5%	
III	16, 14.3%	6, 37.5%	
IV	19, 21.4%	4, 25%	
**+ extranodal localization (n, %)**	11, 15.7%	4, 25%	0.465†
**+ B symptoms (n, %)**	43, 61.4%	5, 68.8%	0.7757†
**+ bulky disease (n, %)**	12, 17.2%	3, 18.8%	0.9999†
**EORCT (for stages I & II, n, %)**			0.5720#
0	13, 28.9%	0, 0%	
1	12, 26.7%	3, 50%	
2	13, 28.9%	3, 50%	
3	7, 15.5%	0, 0%	
**GHSG (for stages I & II, n, %)**			0.3988#
0	12, 26.7%	0, 0%	
1	13, 28.9%	2, 33.3%	
2	14, 31.1%	4, 66.7%	
3	6, 13.3%	0, 0%	
**IPS (for stages III & IV, n, %)**			0.8669#
1	5, 20%	3, 30%	
2	9, 36%	1, 10%	
3	7, 28%	4, 50%	

4	2, 8%	0, 0%	
5	2, 8%	1, 10%	
**ECOG (n, %)**			0.5723#
0	53, 75.7%	11, 68.7%	
1	13,18.6%	5, 31.3%	
2	4, 5.7%	0, 0%	
**Response to 1st line of CTx. (n, %)**			0.7704†
CR	60, 87%	11, 85%	
PR	6, 8.7%	0, 0%	
PD	3, 4.3%	2, 15%	

* t- test, † Fisher's exact test, # Mann - Whitney test

Abbreviations used are: SATB1-, SATB1 negative; SATB1+, SATB1 positive; IQR, interquartile range; n, number of cases; EORCT, European Organization for the Research and Treatment of Cancer; GHSG, German Hodgkin's Study Group; IPS, International Prognostic Score; ECOG, Eastern Cooperative Oncology Group; CTx, conventional chemotherapy; CR, complete response; PR, partial response; PD, progressive disease.

Table S2. p16 expression level correlation with clinicopathological characteristics of HL patients at diagnosis and response to first line of chemotherapy.

	**p16-**	**p16+**	**p value**
**number of patients**	74	12	
**age (years, median, IQR)**	42 (28.7 to 56.8)	31.5 (20.7 to 52)	0.1653*
**sex (n, %)**			0.9999†
female	34, 49%	5, 41.7%	
male	40, 54%	7, 58.3%	
**histological type (n, %)**			0.9999†
mixed cellularity	17, 22.9%	3, 25%	
nodular sclerosis	52, 71.4%	8, 62.5%	
other	5, 5.7%	1, 12.5%	
**clinical stage (n, %)**			0.5725†
I	9, 12.1%	0, 0%	
II	35, 47.3%	7, 58.3%	
III	14, 18.9%	2, 16.7%	
IV	16, 21.6%	3, 16.7%	
**+ extranodal localization (n, %)**	13, 15.7%	2, 25%	0.9999†
**+ B symptoms (n, %)**	47, 63.5%	7, 58.3%	0.7553†
**+ bulky disease (n, %)**	12, 16.2%	3, 25%	0.4317†
**EORCT (for stages I & II, n, %)**			0.6884#
0	11, 25%	2, 28.6%	
1	13, 29.5%	2, 28.5%	
2	13, 29.5%	3, 42.8%	

3	7, 15.9%	0, 0%	
**GHSG (for stages I & II, n, %)**			0.9454#
0	10, 22.7%	2, 28.6%	
1	14, 31.8%	1, 14.3%	
2	14, 31.8%	4, 57.1%	
3	6, 13.7%	0, 0%	
**IPS (for stages III & IV, n, %)**			0.3961#
1	6, 20%	2, 40%	
2	9, 30%	1, 20%	
3	10, 33.3%	2, 40%	
4	2, 6.7%	0, 0%	
5	3, 10%	0, 0%	
**ECOG (n, %)**			0.4619#
0	54, 72.9%	10, 83.3%	
1	16, 21.6%	2, 16.7%	
2	4, 5.4%	0, 0%	
**Response to 1st line of CTx. (n, %)**			0.7527†
CR	61, 87.1%	10, 83.3%	
PR	3, 4.3%	0, 0%	
PD	6, 8.6%	2. 16.7%	

* T-test, † Fisher's exact test, # Mann-Whitney

Abbreviations used are: p16-, p16 negative; p16+, p16 positive; n, number of patients; IQR, interquartile range; n, number of cases; EORCT, European Organization for the Research and Treatment of Cancer; GHSG, German Hodgkin's Study Group; IPS, International Prognostic Score; ECOG, Eastern Cooperative Oncology Group; CTx, conventional chemotherapy; CR, complete response; PR, partial response; PD, progressive disease.

Table S3. Statistical analysis of the correlation of clinicopathological characteristics and three subpopulations of patients based on SATB1 and p16 expression.

	**SATB1+/p16-**	**SATB1+ or -/p16+**	**SATB1-/p16+**	**p value**
**number of patients**	12	12	62	
**age (years, median, IQR)**	44 (30 to 64.8)	31.5 (20.8 to 52)	42 (28 to 53.8)	0.3380*
**sex (n, %)**				0.1058*
female	9, 75%	5, 41.7%	25, 45.3%	
male	3, 25%	7, 58.3%	37, 54.7%	
**histological type (n, %)**				0.9867†
mixed cellularity	2, 16.7%	3, 25%	15, 24.2%	
nodular sclerosis	9, 75%	8, 66.7%	43, 69.4%	

other	1, 8.3%	1, 8.3%	4, 6.4%	
**clinical stage (n, %)**				0.5722†
I	0, 0%	0, 0%	9, 14.5%	
II	5, 41.7%	7, 58.3%	30, 48.5%	
III	4, 33.3%	2, 16.7%	10, 18.6%	
IV	16, 25%	3, 25%	13, 22.1%	
**+ extranodal localization (n, %)**	3, 25%	2, 16.7%	10, 16.1%	0.7413†
**+ B symptoms (n, %)**	9, 75%	7, 58.3%	38, 62.8%	0.7691†
**+ bulky disease (n, %)**	2, 16.7%	3, 25%	10, 16.1%	0.7413†
**EORCT (for stages I & II, n, %)**				0.8872#
0	0, 0%	2, 28.6%	11, 28.2%	
1	3, 60%	2, 28.6%	10, 25.6%	
2	2, 40%	3, 42.86%	11, 28.2%	
3	0, 0%	0, 0%	7, 17.8%	
**GHSG (for stages I & II, n, %)**				0.8121#
0	0, 0%	2, 28.6%	10, 25.6%	
1	2, 40%	1, 14.3%	12, 30.7%	
2	3, 60%	4, 57.1%	11, 28.2%	
3	0, 0%	0, 0%	6, 15.4%	
**IPS (for stages III & IV, n, %)**				0.5145#
1	1, 14.3%	2, 40%	5, 21.7%	
2	1, 14.3%	1, 20%	8, 34.8%	
3	4, 57.1%	2, 40%	6, 26.1%	
4	0, 0%	0, 0%	2, 8.7%	
5	1, 14.3%	0, 0%	2, 8.7%	
**ECOG (n, %)**				0.6609#
0	8, 66.7%	10, 83.3%	46, 74.2%	
1	4, 33.3%	2, 16.7%	12, 19.4%	
2	0, 0%	0, 0%	4, 6.5%	
**Response to 1st line of CTx. (n, %)**				0.3365†
CR	7, 77.8%	10, 83.3%	54, 88.5%	
PR	0, 0%	0, 0%	3, 4.9%	
PD	2, 22.2%	2, 16.7%	4, 6.6%	

* ANOVA, †Fisher's exact tests, # Kruskal Wallis test

Abbreviations used are: SATB1-, SATB1 negative; SATB+, SATB positive; p16-, p16 negative; p16+, p16 positive; n, number of patients; EORCT, European Organization for the Research and Treatment of Cancer; GHSG, German Hodgkin's Study Group; IPS, International Prognostic Score; ECOG, Eastern Cooperative Oncology Group; CTx, conventional chemotherapy; CR, complete response; PR, partial response; PD, progressive disease.
